# Supplementary material for: Measuring uncertainty in human visual segmentation
Source: PLoS Comput Biol. 2023 Sep 25;19(9):e1011483. doi: 10.1371/journal.pcbi.1011483 (PMC10553811; doi:10.1371/journal.pcbi.1011483)
Supplement: S1 Text — Appendix A. Proof of Proposition 1. Appendix B. Stimulus generation. Appendix C. Large or unknown number of segments. Appendix D. Resolution of the segmentation maps. Appendix E. Individual entropy maps. Fig A. Large Number of Segments. To test the feasibility of the reconstruction for a large number of segments, we generated an artificial segmentation map with K = 9 segments and N = 25. The reconstruction obtained from measuring a single repetition of the minimal set of pairs, remains accurate when using spatial regularization. Top: ground truth. Center: no regularization. Bottom: Laplacian regularization. Fig B. Unknown Number of Segments. Reconstruction using different values of K with regularization. Top: ground truth. Then, from top to bottom, reconstruction with K = 3, 4, 5, 6 and 7. If the true K is unknown, it can be correctly inferred from the reconstructed maps, as the maximum value of K that produces no empty maps. Fig C. Resolution. Effect of increases in resolutions over the reconstruction of probabilistic segmentation maps. Top-left: ground truth maps. Top-right: reconstruction without regularization. Bottom-right: reconstruction with Laplacian regularization. MAE between the reconstructed maps and ground truth is indicated on top of each collection of maps. Bottom-left : MAE between the reconstructed maps and ground truth as a function of the resolution. Shaded areas represent 95% bootstrap error bars. Fig D. Wider Kernel Regularization. Effect of the kernel width used for the regularization. This must be compared to Fig C bottom-right. Fig E. Individual entropy maps. Top-left: the 15 participants in the low uncertainty condition. Bottom-left: the 15 participants in the high uncertainty condition. The contour drawn in red is drawn by the participant. Bottom-right: distribution of the contour f-scores of the participants. Table A. Summary of the stimulus parameters. Parameters of the stimuli used in the experiments. (PDF) [file pcbi.1011483.s001.pdf]

Measuring uncertainty in human visual segmentation

Jonathan Vacher[1*¤], Claire Launay[2], Pascal Mamassian[1‡], Ruben Coen-Cagli[2,3,4*‡]

1 Laboratoire des systèmes perceptifs, Département d'études cognitives, École normale supérieure, PSL University, CNRS, Paris, France
2 Department of Systems and Computational Biology, Albert Einstein College of Medicine, Bronx, New-York, United States of America
3 Dominick P. Purpura Department of Neuroscience, Albert Einstein College of Medicine, Bronx, New-York, United States of America
4 Department. of Ophthalmology and Visual Sciences, Albert Einstein College of Medicine, Bronx, New-York, United States of America

‡These authors also contributed equally to this work.
¤Current Address: Université Paris Cité, CNRS, MAP5, Paris, France
* jonathan.vacher@u-paris.fr
* ruben.coen-cagli@einsteinmed.edu

Supporting information

Appendix A. Proof of Proposition 1

Proof. The negative log-likelihood writes

$$\ell((p_{\mathbf{i}})_{\mathbf{i}\in\mathcal{I}};\mathcal{D}_{N_b}) = -\sum_{n=1}^{N_b}\sum_{(\mathbf{i},\mathbf{j})\in\mathcal{P}_n} r_{\mathbf{i},\mathbf{j}}^{(n)}\log\left(p_{\mathbf{i}}\cdot p_{\mathbf{j}}\right) + (1-r_{\mathbf{i},\mathbf{j}}^{(n)})\log\left(1-p_{\mathbf{i}}\cdot p_{\mathbf{j}}\right).$$

And, its gradient with respect to $p_{\mathbf{u}}$ writes

$$\begin{aligned}\nabla_{p_{\mathbf{u}}}\ell((p_{\mathbf{i}})_{\mathbf{i}\in\mathcal{I}};\mathcal{D}_{N_b}) &= -\sum_{n=1}^{N_b}\sum_{\mathbf{j}|(\mathbf{u},\mathbf{j})\in\mathcal{P}_n}\left(\frac{r_{\mathbf{u},\mathbf{j}}^{(n)}}{p_{\mathbf{u}}\cdot p_{\mathbf{j}}}-\frac{(1-r_{\mathbf{u},\mathbf{j}}^{(n)})}{1-p_{\mathbf{u}}\cdot p_{\mathbf{j}}}\right)p_{\mathbf{j}}\\ &= -\sum_{\mathbf{j}|(\mathbf{u},\mathbf{j})\in\mathcal{P}}\sum_{n=1}^{N_{\mathbf{u},\mathbf{j}}}\left(\frac{r_{\mathbf{u},\mathbf{j}}^{(n)}}{p_{\mathbf{u}}\cdot p_{\mathbf{j}}}-\frac{(1-r_{\mathbf{u},\mathbf{j}}^{(n)})}{1-p_{\mathbf{u}}\cdot p_{\mathbf{j}}}\right)p_{\mathbf{j}}\\ &= -\sum_{\mathbf{j}|(\mathbf{u},\mathbf{j})\in\mathcal{P}}\left(\frac{k_{\mathbf{u},\mathbf{j}}}{p_{\mathbf{u}}\cdot p_{\mathbf{j}}}-\frac{(1-k_{\mathbf{u},\mathbf{j}})}{1-p_{\mathbf{u}}\cdot p_{\mathbf{j}}}\right)N_{\mathbf{u},\mathbf{j}}p_{\mathbf{j}},\end{aligned}$$

where for all $(\mathbf{i},\mathbf{j})\in\mathcal{I}^2$, $k_{\mathbf{i},\mathbf{j}}=\frac{\sum_{n=1}^{N_b} r_{\mathbf{i},\mathbf{j}}^{(n)}}{N_{\mathbf{i},\mathbf{j}}}$. Similarly, the least-square loss writes

$$\ell_s\left((p_{\mathbf{i}})_{\mathbf{i}\in\mathcal{I}};\mathcal{D}_{N_b}\right) = \sum_{(\mathbf{i},\mathbf{j})\in\mathcal{P}}\|k_{\mathbf{i},\mathbf{j}}-p_{\mathbf{i}}\cdot p_{\mathbf{j}}\|^2.$$

And, its gradient with respect to $p_{\mathbf{u}}$ writes

$$\nabla_{p_{\mathbf{u}}}\ell_s((p_{\mathbf{i}})_{\mathbf{i}\in\mathcal{I}};\mathcal{D}_{N_b}) = 2\sum_{\mathbf{j}|(\mathbf{u},\mathbf{j})\in\mathcal{P}} p_{\mathbf{j}}(k_{\mathbf{u},\mathbf{j}}-p_{\mathbf{u}}\cdot p_{\mathbf{j}})$$

Then, the vectors $(p_{\mathbf{j}})_{\mathbf{j},(\mathbf{u},\mathbf{j})\in\mathcal{P}}$ being linearly independent and a sub-family of a critical point of ℓ and ℓ_s, we have the following equivalences

$$\begin{aligned}\forall \mathbf{u}\in\mathcal{I},\ \nabla_{p_{\mathbf{u}}}\ell((p_{\mathbf{i}})_{\mathbf{i}\in\mathcal{I}};\mathcal{D}_{N_b})=0 \quad &\iff \quad \forall(\mathbf{i},\mathbf{j})\in\mathcal{I}^2,\ \frac{k_{\mathbf{u},\mathbf{j}}}{p_{\mathbf{u}}\cdot p_{\mathbf{j}}}-\frac{(1-k_{\mathbf{u},\mathbf{j}})}{1-p_{\mathbf{u}}\cdot p_{\mathbf{j}}}=0\\ &\iff \quad \forall(\mathbf{i},\mathbf{j})\in\mathcal{I}^2,\ p_{\mathbf{i}}\cdot p_{\mathbf{j}}=k_{\mathbf{i},\mathbf{j}}\\ &\iff \quad \forall \mathbf{u}\in\mathcal{I},\ \nabla_{p_{\mathbf{u}}}\ell_s((p_{\mathbf{i}})_{\mathbf{i}\in\mathcal{I}})=0.\end{aligned}$$

□

Appendix B. Stimulus generation

The stimuli are generated in two steps:

(i) the generation of the probabilistic segmentation maps,

(ii) the synthesis of the textures composing the image.

The code is provided along with the `vseg` package.

Generation of probabilistic segmentation maps The grid is defined as it follows

$$\mathcal{I}=\begin{cases}\{-\frac{N-1}{2},\dots,\frac{N-1}{2}\}^2 & \text{if } N \text{ is odd},\\ \{-\frac{N}{2},\dots,\frac{N}{2}-1\}^2 & \text{if } N \text{ is even}\end{cases}.$$

Concisely, a probabilistic segmentation map with K segments is generated by exponentiating K independent stationary Gaussian random fields (white noises smoothed by a kernel) and normalizing them so that they sum to one. We write for all $k\in\{1,\dots,K\}$ and all $\mathbf{i}\in\mathcal{I}$,

$$p_{\mathbf{i}}[k]=\frac{e^{f_{\mathbf{i}}[k]}}{\sum_{l=1}^{K}e^{f_{\mathbf{i}}[l]}}$$

where $f_{\mathbf{i}}[k]=(G^{(\sigma,\xi)}*N[k])_{\mathbf{i}}$ with $G^{(\sigma,\xi)}_{\mathbf{i}}=\sigma^2e^{-\|\mathbf{i}\|^2/2\xi^2}$, $*$ is the discrete convolution as defined in Equation (7) and $N[k]$ is white noise image. The parameter σ controls the amplitude of the Gaussian field and hence the uncertainty of the probabilistic map of each segment (see Fig 5). When σ is large the maps are more likely to be composed of 0 and 1 (*i.e.* low uncertainty). When it is small the maps are more likely to be composed of values around $1/K$ (*i.e.* high uncertainty). The parameter ξ controls the size of the segments in the images. When ξ is large, the segments are large. When ξ is small, the segments are small and can be composed of multiple connected components. In practice, the discrete convolution is performed in the Fourier domain.

Texture synthesis For the experiment involving human participants, we used images composed of two segments that are filled with stationary Gaussian oriented textures [2]. The two probabilistic maps were generated as described above using a high value for σ ensuring 0-1 maps. The maps were then smoothed using convolution with a Gaussian kernel of width 2.5 px. To avoid a sharp transition from one texture to the other, the probabilistic maps were used to weight the orientation of each texture. Therefore in order to generate the textures (see Fig 9), we performed a convolution between a white noise image and a spatially varying kernel parametrized by a local orientation defined by $\theta_{0\mathbf{i}}=p_{\mathbf{i}}[1]\theta_0^{(1)}+p_{\mathbf{i}}[2]\theta_0^{(2)}$. The kernel κ is defined in the Fourier domain with polar coordinates $(r,\theta)\in[0,1/2]\times[0,\pi]$ by

$$\hat{\kappa}(r,\theta)=\exp\left(\frac{\cos\left(2(\theta-\theta_0)\right)}{4\sigma_\theta^2}\right)^{\frac{1}{2}}\exp\left(-\frac{\log\left(r/r_0\right)^2}{2\log\left(1+\sigma_r^2\right)}\right)^{\frac{1}{2}}.$$

In practice, we use the following parametrization

$$\sigma_r = \sqrt{\exp\left(\frac{\ln(2)}{8} B_r^2\right) - 1} \quad \text{and} \quad r_0 = \frac{m_r}{N_{\text{px cm}^{-1}}}(1 + \sigma_r^2)$$

where B_r is a frequency bandwidth (in octave), m_r is the mode of the log-normal distribution and $N_{\text{px cm}^{-1}}$ is the number of pixel per centimeter of the screen used to generate the stimuli. The values of the parameters is summarized in Table A.

	θ_0 (deg)	σ_θ ($\sim$ deg)	m_r (c/ deg)	B_r (oct.)	RMS Constrast (gray lvl)
Low Uncertainty	-5 and 5	5	2.45	2	35
High Uncertainty	-5 and 5	7.5	2.45	2	35

Table A. Summary of the stimulus parameters.

Appendix C. Large or Unknown Number of Segments

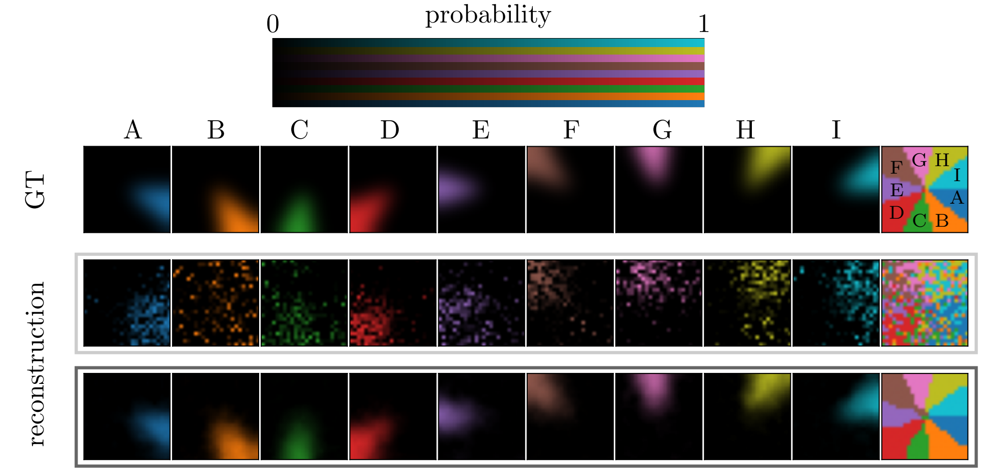

Fig A. Large Number of Segments To test the feasibility of the reconstruction for a large number of segments, we generated an artificial segmentation map with $K = 9$ segments and $N = 25$. The reconstruction obtained from measuring a single repetition of the minimal set of pairs, remains accurate when using spatial regularization. Top: ground truth. Center: no regularization. Bottom: Laplacian regularization.

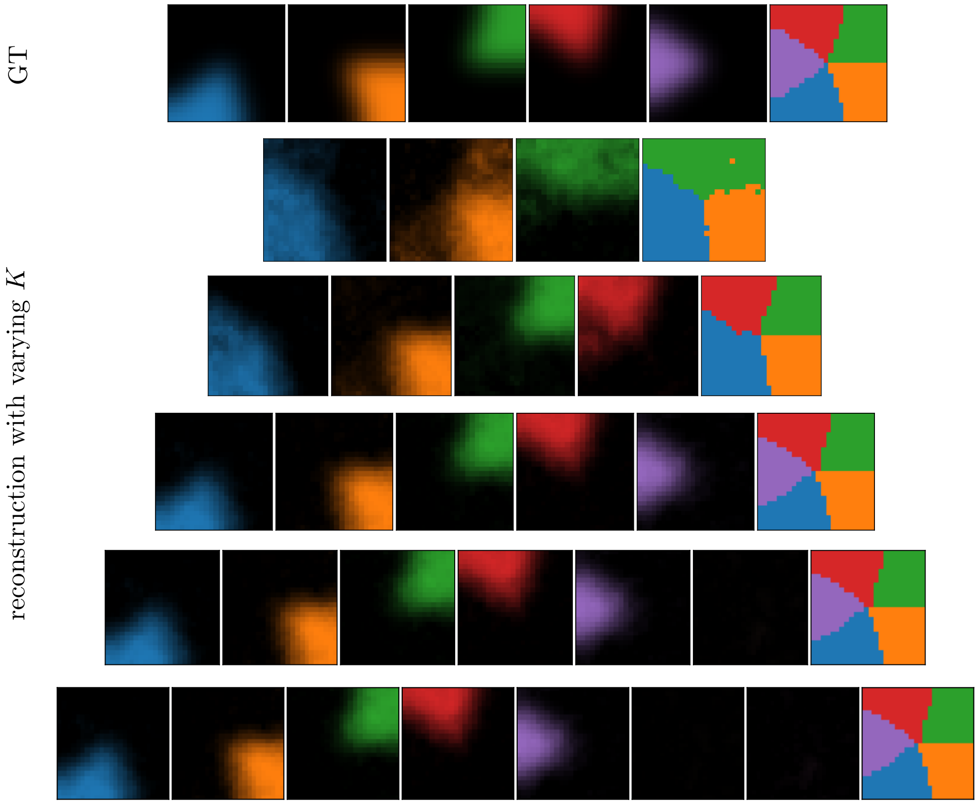

Fig B. Unknown Number of Segments Reconstruction using different values of K with regularization. Top: ground truth. Then, from top to bottom, reconstruction with $K = 3, 4, 5, 6$ and 7. If the true K is unknown, it can be correctly inferred from the reconstructed maps, as the maximum value of K that produces no empty maps.

Appendix D. Resolution of the segmentation maps

The minimum number of pairs that need to be tested to enable reconstruction (see section *Material and Methods*) scales quadratically with the grid size N of segmentation map . Because in real experiments the number of pairs one can test is limited, to guide experimental design here we use simulations to explore the effects of varying the grid size relative to the resolution of the input image.

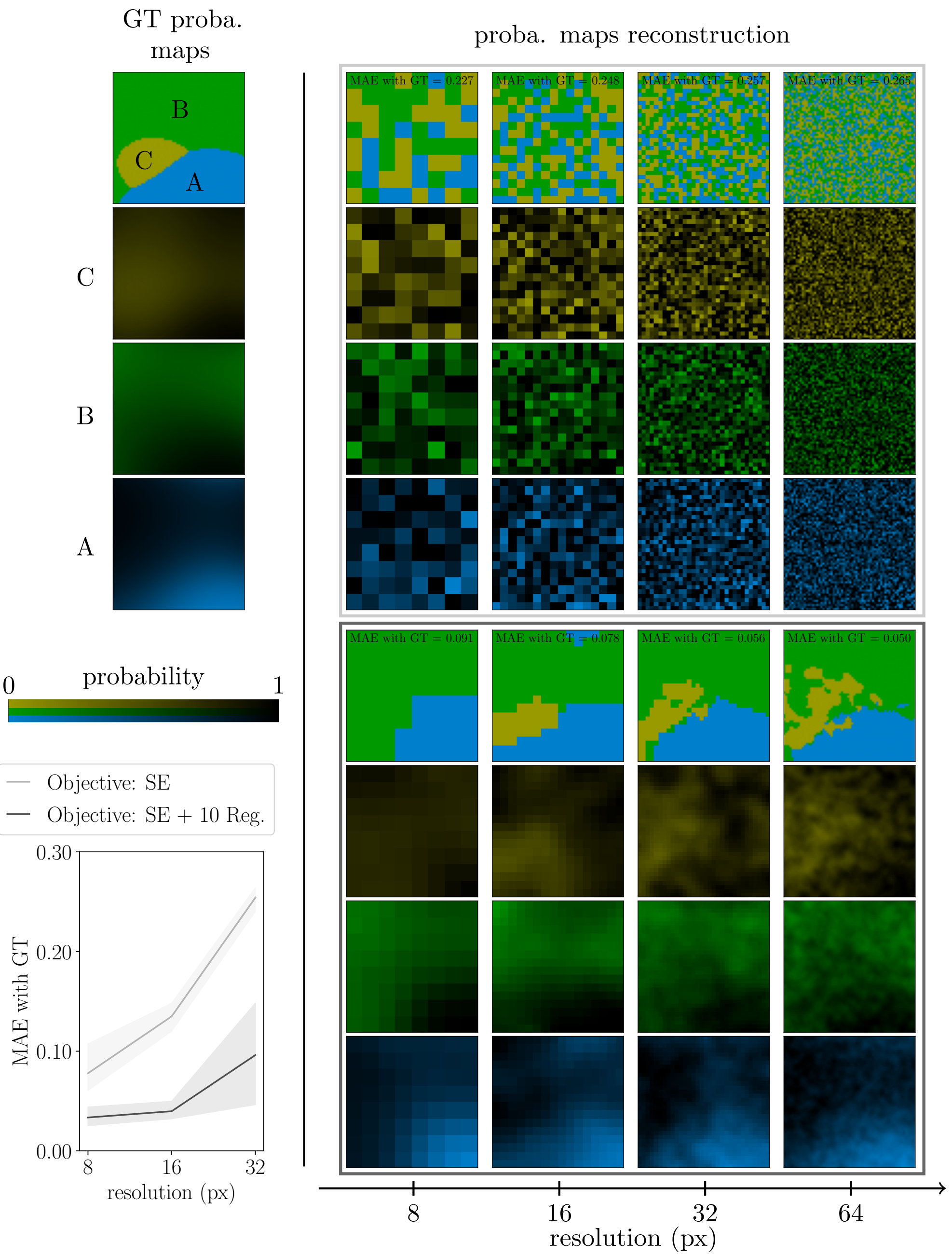

Fig C. Resolution Effect of increases in resolutions over the reconstruction of probabilistic segmentation maps. Top-left: ground truth maps. Top-right: reconstruction without regularization. Bottom-right: reconstruction with Laplacian regularization. MAE between the reconstructed maps and ground truth is indicated on top of each collection of maps. Bottom-left : MAE between the reconstructed maps and ground truth as a function of the resolution. Shaded areas represent 95% bootstrap error bars.

In order to test the robustness to varying the grid size, we generated an artificial segmentation map with grid size $N = 64$ and we simulated data from the sub-sampled maps with sizes $N = 8, 16, 32$ and 64. In a real experiment, this is analogous to showing

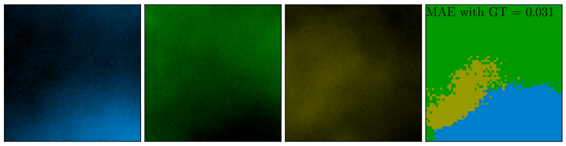

Fig D. Wider Kernel Regularization Effect of the kernel width used for the regularization. This must be compared to Fig C bottom-right.

to the participants the image at full resolution ($N = 64$) and reconstructing the maps at different resolutions (*i.e.* testing different numbers of pairs). Fig C illustrates that, for this specific example, spatial regularization allows to recover the probabilistic segmentation maps accurately ($< 10\%$ MAE) at all resolutions, while in the absence of spatial regularization the inference only recovers noise ($\sim 25\%$ MAE; notice that here the ground-truth maps have much higher uncertainty compared to Fig 4, hence the poorer performance). When using spatial regularization the inference can miss segments which have a small area (e.g. the case with lowest resolution in Fig C). Due to the locality of the Laplacian, the reconstruction appears better at low to intermediate resolutions than at high resolutions. As illustrated by the bottom-left graph, the Laplacian regularized reconstructions are more robust than the unregularized ones. Indeed, the MAE of the regularized reconstruction is much lower compared to the unregularized ones. The MAE also increases slower as the resolution increases when regularization is used than when it is not. In addition, the increase in MAE for the regularized maps can be corrected by using a wider regularization kernel G, see equation (7). Indeed, the kernel width used for regularization has not been adjusted to the image resolution in Fig C compared to the result showed in Fig D. Though, we do not expand more on this issue because high resolutions are still inaccessible in our current experimental settings (it requires too many trials).

Appendix E Individual entropy maps

The data collected using synthetic textures allowed us to infer the probabilistic maps for each participant and to compute their corresponding entropy map shown in Fig E (the average is shown in Fig 7). We observe that entropy appears higher for more participants in the high uncertainty condition than in the low uncertainty condition (summarized by the t-test in Fig 7). This corroborates another qualitative observation that the contours drawn by the participants are more variable for the high uncertainty condition (9/15 are different from the ground truth) than for the low uncertainty condition (3/15 are different from the ground truth). Such an observation is quantitatively reflected in the contour f-score [1] of the participants. In the low uncertainty condition, contours are more consistent with the ground truth contour (high f-score) than in the high uncertainty condition (low f-score). The same observation holds when computing inter-participants f-score (not shown), reported contours are more consistent across participant in the low uncertainty condition than in the high uncertainty condition.

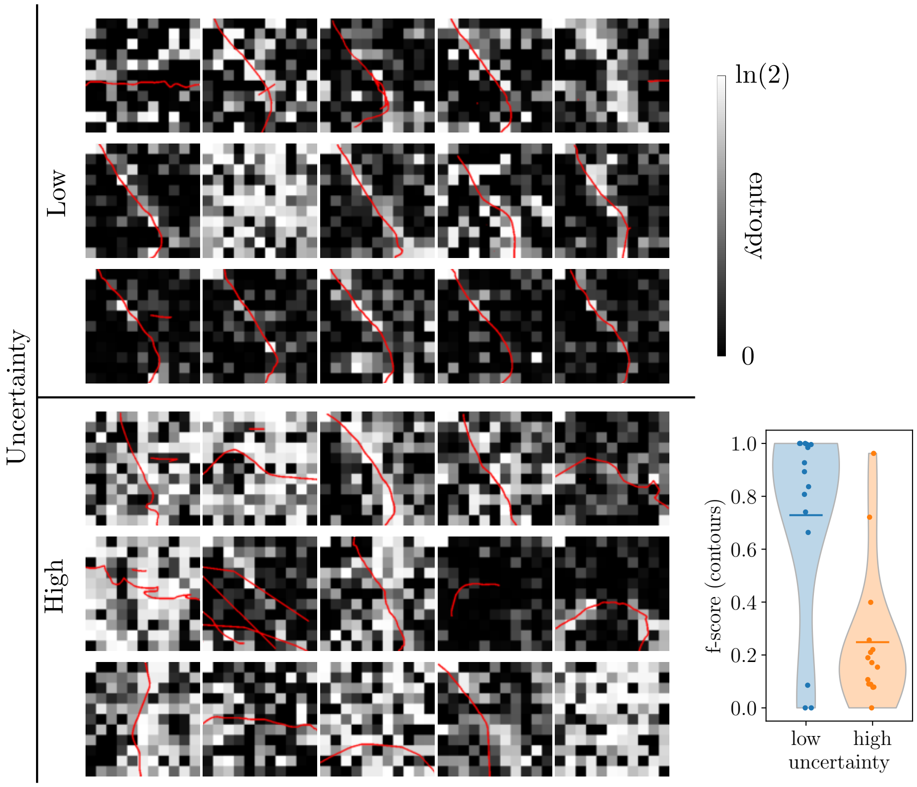

Fig E. Individual entropy maps Top-left: the 15 participants in the low uncertainty condition. Bottom-left: the 15 participants in the high uncertainty condition. The contour drawn in red is drawn by the participant. Bottom-right: distribution of the contour f-scores of the participants.

References

1. Arbelaez P, Maire M, Fowlkes C, Malik J. Contour detection and hierarchical image segmentation. IEEE transactions on pattern analysis and machine intelligence. 2011;33(5):898–916.

2. Vacher J, Meso AI, Perrinet LU, Peyré G. Bayesian modeling of motion perception using dynamical stochastic textures. Neural computation. 2018;30(12):3355–3392. doi:10.1162/neco_a_01142.
