# Supplementary material for: Measuring uncertainty in human visual segmentation
Source: PLoS Comput Biol. 2023 Sep 25;19(9):e1011483. doi: 10.1371/journal.pcbi.1011483 (PMC10553811; doi:10.1371/journal.pcbi.1011483)
Supplement: Attachment — Submitted filename: response_to_reviewers.pdf [file pcbi.1011483.s003.pdf]

Jonathan Vacher
Laboratoire MAP5 UMR 8145,
Université de Paris,
UFR Mathématiques & Informatique,
Campus Saint-Germain-des-Prés,
45 rue des Saints-Pères, 75006 PARIS

June 29, 2023

Dear Editor of PLoS Computational Biology journal and reviewers,

We would like to thank the editor for accepting to send our manuscript for review and both reviewers for their detailed feedback. We appreciate that both reviewers positively judged our manuscript. We have performed substantial additional analyses and edited the manuscript accordingly(see enclosed files, with modified text in red), as described in the detailed response letter . To summarize, we have addressed the major criticisms related to the following topics:

- Intra-participant variability (both reviewers): we have clarified that our approach is the first to measure perceptual variability for individual participants, and added a new Figure 14 showing single-participant variability maps for all participants.
- Limitations of existing manual datasets (reviewer 2): we have added detailed explanations; provided in Fig 6 and 14 the manual segmentations obtained from our participants, for reference; and provided a figure comparing segmentation maps from our participants to those of the BSD dataset, in the rebuttal letter.
- Duration of experiments (reviewer 2): we provide more detailed estimates of duration, and the measured response times (Fig 1 in the rebuttal letter), showing that multiple images can be segmented in a 2-hour session. We add broader motivation and literature for why long sessions are valuable for studying perception. We also show in the rebuttal letter one successful approach to substantially reduce the duration (parametric reconstruction; Fig 2).
- Clarity and mathematical details (reviewer 1): we have followed all the suggestions.
- We also addressed all other details (both reviewers).

We believe that we have sufficiently addressed the reviewers' concerns and we hope it is now ready for acceptance.

Sincerely,

Jonathan Vacher and co-authors

Encl: Detailed responses to reviewers. Edited manuscript with highlights. Edited manuscript.

Response to reviewer 1

Rev 1 : This paper describes a technique for determining perceived image segmentation on a pixel-by-pixel (or rather, grid-block-by-grid-block) basis using choice data. It is a substantial contribution and should be published. I have some substantive comments below, but they are all easily fixable. My main kvetch is that the math is needlessly obscure and could use some clarity to make it easier to follow.

We thank the reviewer for the detailed reading and feedback of our article. We appreciate the acknowledgment that our work provides a substantial contribution and that it should be published. We have addressed all the comments and suggestions in the revised manuscript, as detailed below.

Rev 1 : OK, this one isn't by line number. Decades ago, my friend Pete Mowforth (once a co-founder of an image-processing group in Glasgow called the Turing Institute) sent out a natural image to a bunch of well-known researchers in image processing and computer vision (think: people on the order of Azriel Rosenfeld) and asked them to mark where the edges were (not identical to asking for image segmentation, but close). The results were wildly variable across people, with some indicating any color or luminance boundary, and others indicating object boundaries only, including completion of boundaries through locations where no actual luminance/color boundary was in the image, etc. The point is that "segmentation" is necessarily multi-scale and ill-defined. Yet, the main work here involves combining data across participants and there really isn't enough discussion about the importance and meaning of inter-participant differences in strategy in this task.

Thank you for sharing this interesting anecdote. Based on this and similar comments from R2, we realize that the way we presented our data was misleading, and we have now corrected this issue. The main point of the proposed method is that it measures the perceptual segmentation map and its uncertainty for individual participants. In the original manuscript we showed only figures with aggregate analyses. Now, we have added Figure 14 and text (S5 Appendix) that show the uncertainty maps of individual participants.

However, we agree that segmentation is indeed ill-defined, and that there are many possible factors of variations between participants. Our proposal of a standardized protocol is a way to better control for at least some of these factors, as we explain in the revised text (Introduction lines 39-44). We also believe that our method could enable future work to further manipulate some of these factors, using small variations of our task (*e.g.* masking most of the image except some small area around the two cues; giving more "bottom-up" instructions; or cueing specific objects).

Rev 1 : 111-116: This is about the first time (well, slightly above) that we see the word "grid" and the value of N is given even though N has not yet been defined. So, clarify that you'll be asking for judgments on a square grid with a resolution coarser than the pixel resolution in the images. Clarify somewhere where the red dot is placed within the grid square. Clarify why $N_t = KN^2$. At this point, I'd think that there are N^2 grid squares and you are testing arbitrary pairs, so that the number of points tested should be on the order of N^2-choose-2 (i.e., on the order of N^4) and that the value of K isn't relevant. So, this definition of N_t comes out of the blue here. And why does the number tested (per block) drop to $(K-1)N^2$ 5 lines later?

Thank you for pointing out these inconsistencies. We have now explained clearly the selection of points on the grid (lines 122-127), reported more clearly the choice of N_t for the experiments we conducted (Paragraph *Detailed choices for each experiment*), and added a pointer in the text where the minimum number of trials is explained.

Rev 1 : 173: The "grid" is defined to be "script-I" on 158 and yet here you state that the pairs are drawn from script-I^2. And, you now act like script-I isn't the grid (the set of grid

squares), but rather the pixels inside those grid squares that will be tested. Unclear.

We have revised the text to clarify it as follows (lines 203-206). The notation $\mathcal{I}$ for the grid, indicates the set of x, y coordinates of the centers of all the elements of the grid (each element is a square, if the image length and width are equal, as in all of our experiments). The notation $\mathcal{I}^2$ indicates the set of the coordinates of all the pairs of points (x_1, y_1 and x_2, y_2). Therefore, a single point on the grid is drawn from $\mathcal{I}$, and a pair of points is drawn from $\mathcal{I}^2$.

Rev 1 : Eq. 1: This is the most needlessly obscure and, frankly, incorrect piece of the math. Anyone who has coded a psychometric function fit would know that the likelihood of a single trial is either p or 1-p, depending on the response. Not everyone has seen the shorthand of coding the likelihood as $p^r(1-p)^{(1-r)}$, and this shorthand depends on coding r as 0 or 1, which you never state overtly. Finally, stating the log of this as the sum of KL distance and cross entropy is both unnecessary and incorrect. "r" is not a probability, it's an actual response. Its value is zero or one, so you can't take one or the other log in the definition of KL. Sure, if you act like you can take this log and write out the sum of KL and H, terms cancel and you end up with the correct expression. But why do this?

We have simplified eq. 1 according to the suggestion, that we agree avoids unnecessary complications. We would like to point out that in the original version, the mathematics underlying Equation 1 were correct because the function $x \mapsto x \log(x) + (1-x)\log(x)$ can be extended by continuity at 0 and 1 and so the KL divergence is always well defined.

Rev 1 : 185: Here's my main confusion with the entire paper: I'm not sure what it means that the segment assignments are "independent". I think what you mean, and this should be clarified, is that when an observer, whom we are modeling with these k-tuples of probabilities per grid square, is asked about a pair, the observer gets an assignment for each element of the pair, drawn from the multinomial indicated by the grid squares k-tuple, and the draws for the two grid squares are independent. That's what is required for Eq. 2 to be true. I got distracted by the idea that where the probability assignments came from was independent or some such obscure complexity. So, clarify that what you mean is actually about your model of the observer.

We have revised the text around lines 225-227 to clarify. Your first intuition is the correct one, the segment assignment of pixels are independent. In terms of probabilities this means that for each pixel, we have a random variable $C_\mathbf{i}$ (the segment label of pixel $\mathbf{i}$, which can be any integer between 1 and K) that depends on $p_\mathbf{i}$. For all pairs of pixels $(\mathbf{i}, \mathbf{j})$ with $\mathbf{i} \neq \mathbf{j}$, the random variables $C_\mathbf{i}$ and $C_\mathbf{j}$ are independent conditionally on $p_\mathbf{i}$ and $p_\mathbf{j}$ respectively. We clarify this in the text without introducing those extra random variables $C_\mathbf{i}$.

Rev 1 : Throughout, there's the usual issue with k-means and other clustering algorithms that the cluster "names" can be permuted and fit just as well. Here, the assignments of segments to the K labels is arbitrary and so multiple runs will likely land on different permutations but be otherwise equivalent. Worth a comment/clarification?

We have added a corresponding statement before proposition 1.

Rev 1 : 194: l_0 is MINIMIZED when...

Fixed, thanks!

Rev 1 : 199-200: Here is a good example of why I'm still confused about what you mean by "independent". You say the family is independent, but that sounds like you are treating the p's as random variables, rather than treating the grid squares' assignments (based on the p's) as random multinomial variables. This phrasing leads me astray.

Absolutely, we apologize for the confusion. Here, we are talking about linear independence (*i.e.* in a deterministic sense). We added the word "linearly" in the proposition.

Rev 1 : Proposition 1: Again the notation is needlessly obscure, especially the use of the indicator function. Somewhere you should say in plain English what these things are (the $k_{i,j}$'s

are just the proportion of same-segment responses for pair i, j and the formula is computing squared error relative to the predicted probabilities of those responses; $N_{i,j}$ is just the number of times i, j was tested and from your description earlier, that shouldn't depend on the pair i, j, I'd think.

We have simplified accordingly. In general the number $N_{i,j}$ does depend on i, j because the set of pixel pairs that can be tested at each block can vary. But in this paper, we always test the same pairs across blocks, so the reviewer is correct that $N_{i,j}$ does not depend on the pair.

Rev 1 : A minor harumph: I generally disklike it when a paper invents a bunch of non-standard acronyms and expects the reader to remember them all. The is worse when those acronyms might already have a meaning in the reader's head. I tend to read SE as standard error, MAE as motion after-effect, BCE as "before the common era" (politically correct for BC) and GT as "greater than". Bleccchhhh. BCE is particularly bad, since its meaning is an obscurity to begin with rather than simply referring to log likelihood. MAE is also confusing since it's defined on pairs of 3-tuples, so how do you turn that into scalar error?

Unfortunately this is indeed a challenge when writing a paper that is (hopefully) of interest to readers in different disciplines. Binary Cross Entropy is the name given in pytorch (and in the machine learning community, more broadly) to the likelihood of a Bernoulli random variable, we have kept this name so that is easier for the reader to match the maths as detailled in the paper and in the code.

About MAE, we decided to introduce this acronym because it sounded more intuitive than "mean L^1 error". We have added text (line 267) with the explicit definition and stating why it is important: "The MAE is defined as the L^1 norm of the differences between the K-tuple of the ground truth and reconstructed probability at each pixel, averaged over pixels. Because it is measured on the probabilistic maps, the MAE reflects the accuracy of the estimation of uncertainty."

Rev 1 : Fig. 2, lower-left panel: Do you ever remark on the fact that the unregularized MAEs get worse as the iterations proceed? Is MAE the right way to think about error anyway, since it depends only on the two outliers?

As explained above, we have added more details about MAE in the revision. In particular, the MAE was wrongly defined as "Maximum Absolute Error" while it is in fact the "Mean Absolute Error". The MAE measures how well the underlying probabilistic maps match the ground truth. It is in fact not penalized by outliers. It reflects both the noisy aspects of the probabilistic maps and the misassignement of the pixels. We have revised the text (lines 272-276) to address this: "Notice that the MAE increases as the optimization of SE or BCE progresses, confirming the visual impression that both the reconstructed segmentation map and the probability maps are noisy and quite different from the ground truth."

Rev 1 : 247: This is yet again an example that seems to mean a different kind of independence than I thought you meant. So, clearly I remain confused about this.

Here, we are talking about the linear independence assumption of Proposition 1. We added the word "linear" to the text.

Rev 1 : Eq. 7 and the short text afterward: Again, this is just stated in math and not in English. If G is a Gaussian, then you are imposing a cost for p being different than the local average over a neighborhood, which is pretty straightforward. I'm not sure what a Laplacian plus a constant (is δ a constant???) buys you, but I would imagine that i's p-value should be compared to a local average over a neighborhood that omits pixel i. Clarify.

We have simplified the notation, and added the intuitive explanation as suggested by the reviewer (lines 317-320).

Rev 1 : "Classes of a pixel pair are independent": Every time the wording about independence comes up, I only get more confused. Most of these wordings sound like the pixel

K-tuples "p" are random vectors and thus independence for different pixels means they are drawn independently from whatever distribution these K-tuples come from. I doubt you mean that, but I'm lost.

We have simplified the wording to refer to the hypothesis underlying Equation (2). To be sure that everything is clear: in Equation (7) the regularization term weighted by λ is introducing dependencies between the probabilities $(p_{\mathbf{i}})_{\mathbf{i}}$ considered as random variables but not between segment assignments $(C_{\mathbf{i}})_{\mathbf{i}}$.

Rev 1 : 280 through Eq. 10: Again this is needlessly obscure and could be made more readable.

We have simplified the notation.

Rev 1 : 380: ... robustly capture uncertainy if uncertainty is as spatially lowpass as your regularizer/blur kernel.

We added this precision (line 435).

Rev 1 : Around Figure 5: I got the impression that you are getting pretty segmentations with only 2 or 3 segments because of pooling over observers. If some observers have fewer and some have more segments, the pooling will end up being well fit by the lowest number of segments on which the subjects tend to agree. I might be wrong, but I think your result of so few segments likely is a result of this. For example in Figure 6, in the first row I'd imagine some observers might have made the reflection a segment, or in the 2nd the 3 rocks as separate segments, or in the 5th row they may have made separate segments for the two cars, but combining subjects ended up merging these separate "objects".

This comment refers to Figure 6. We also think that this result is due to the pooling over participants. Indeed, in the revised manuscript we have superimposed the contours drawn by the participants and there are contours delimiting objects that are not inferred by our algorithm. Even though this hypothesis should be rigorously tested at some point, we think this is beyond the scope of this paper.

Rev 1 : 432-433: I don't see how these results imply the observers have an estimate of their uncertainty (although I've done my best, in other contexts, to prove just that!).

That is true, we could only state that their behavior correlates with the level of uncertainty that is present in the image. However, the manuscript does not state that our results imply that the observers have an estimate of their uncertainty. We are only stating that our results are consistent with this hypothesis.

Rev 1 : S1, first display equation: Shouldn't the sum go from 1 to N_b, not N_t?

Thanks! Corrected.

Rev 1 : S2, the first display equation and the equations in the line following: I'm lost here as well. Why does the "Gaussian"s exponent have a term $||i||^2$??? The pixel index??? I couldn't clearly parse any of this and the term "Gaussian random field" doesn't help me figure out what the equations are doing.

We have added a definition of the grid and a plain English explanation of what is a Gaussian random field. We hope that this is sufficient to clarify that we are defining K white noise images of size $N \times N$ that we spatially smooth using a Gaussian kernel $G^{(\sigma,\xi)}$. Those smoothed white noise images are then exponentiated and normalized so that they sum to 1.

Response to reviewer 2

Rev 2 : The authors present a novel method for extracting segmentation masks from time-limited human same-different forced choice trials. They show that they are able to reconstruct segmentation maps from such trials reliably using both human psychophysics and simulation,

and they discuss in detail the specific experimental setups and potential methods for reconstructing segmentation masks from human trials. Overall, I believe the work is scientifically solid but I have major concerns about the phrasing of the work, particularly in the introduction, as well as some concern about its usefulness and whether the underlying task structure is any more quantitative than extant segmentation methods. That being said, as the authors point out in the discussion of the paper, there are foreseeable ways to improve the general procedure both conceptually and practically in future work. The current work, to the best of my knowledge, makes the first serious attempt at computing segmentation masks from well-controlled psychophysical experiments and I believe this work could serve as an important starting point for other work to build upon, could lay the groundwork for evaluating the validity of segmentation masks in simple stimuli, as well as provide insight into the human visual system in small-scale experiments.

We thank the reviewer for the thoughtful reading of our manuscript and the positive feedback. We have addressed all the comments and suggestions in the revised manuscript, as detailed below.

Rev 2 : Major comments

Rev 2 : 1. The authors state that there are at least three shortcomings with existing human segmentation databases. While I agree these are all potential problems of extant segmentation databases, I am not convinced that the present method convincingly fixes any of them. Below I list the statements and my responses to them:

a. Existing databases rely on manual tracing of contours, introducing interactions between perceptual processes, motor planning and execution, and motor noise. It seems difficult to me to be convinced that the interaction between perception and motor planning/execution, let alone motor noise, play an important role in current segmentation datasets. This can be quantified: in the Cityscapes segmentation challenge (Cordts et al., 2016), the authors assessed the quality of their labeling by having different annotators label the same images. The images had 98% pixel-level agreement between annotators. This level of agreement suggests a hard upper bound on motor noise, planning and execution that is very low (I suspect much lower than the error imposed by the very low resolution of the perceptually produced segmentation masks in this work).

The reviewer raises an important point, highlighting a lack of clarity in our original manuscript, which we have addressed in the revised text. We would like to distinguish two factors.

1) Motor noise: we agree that this is likely a small contribution to the variability of segmentation masks obtained from manual tracing. We no longer mention motor noise in the revised text.

2) Interaction between perceptual processes and motor planning. We have revised the text around lines 39-44 and lines 573-580 to clarify. What we meant is that this interaction can introduce bias and variability, neither of which reflects perceptual processing per se. First, smooth tracing movements require less effort than discontinuous movements. This fact can bias participants to segment the image using smoother boundaries than perceived. For instance, in manually segmenting a scene with a tree, it is likely that participants will draw the outline of the tree as a single boundary, as opposed to segmenting out each individual leaf and branch, even when leaves and branches are clearly perceptually distinct. Second, this effect can translate into variability between different participants, because their effort level is also likely to vary. To illustrate this, please see some examples from the BSD dataset in Figure 3 of this response letter. In semantic segmentation databases like Cityscapes, it is likely that this variability is reduced because the object classes to be segmented are pre-specified (for instance, because 'tree' is one class, participants are discouraged from segmenting branches/foliage separately).

In our task, the effort required to *report* a perceptual judgment does not depend on the

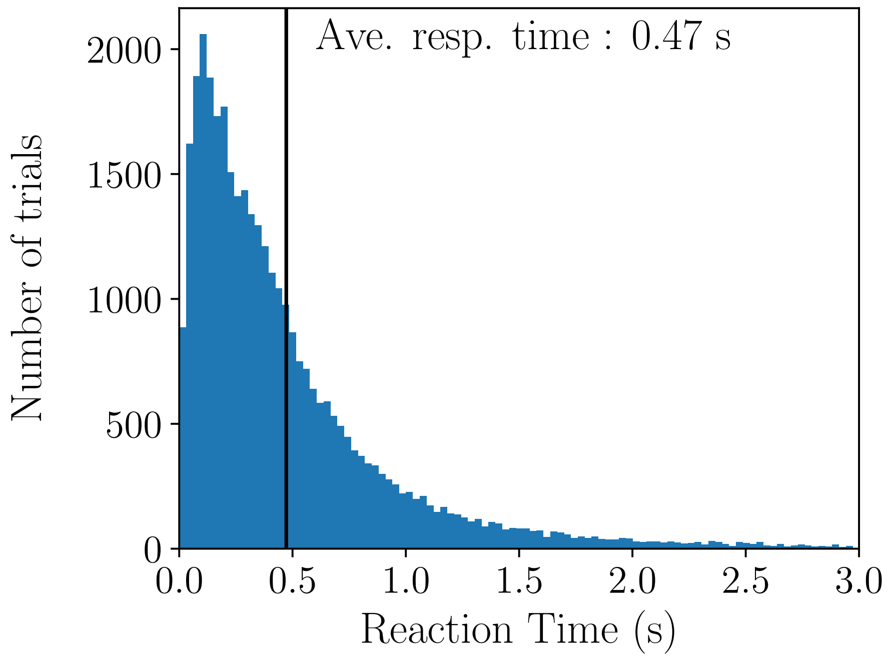

Figure 1: Histogram of the response time of all participants to the experiment involving the segmentation of artificial textures.

smoothness of contours. Removing this confound is important as it can mask perceptual factors of variation, such as whether participants judge two regions as separate segments because they are visually different, or as grouped because they are perceived as parts of a single physical object. We also note that the effort to *reach* that perceptual judgment certainly depends on the visual features (including contour smoothness), and, importantly, we also measure behavioral correlates of that effort, *i.e.* reaction time (see Figure 1) and across-trial variability.

Rev 2 : b. Existing databases have no constraints or measurements of timing. It is true that in most/all segmentation databases there are no controls of timing. This is an issue, but I wonder to what extent this aspect is meaningfully controlled for in the current paper's experiments. Participants are shown the same stimulus in the same location for up to an hour – the demo file attached to the paper leaves me unconvinced that many of the desiderata of time-controlled experiments (confounds of perception, decision making, attention, etc.) are fulfilled. That being said, I do foresee how it could be if the number of trials needed per image was lowered by a meaningful margin. If this was possible, one could randomly show any of a number of images to a participant, potentially mitigating at least some of these top-down effects that may be present in the current paradigm.

We thank the reviewer for raising these important points and for the suggestion. We have revised the text to improve clarity about the control of timing in our experiment, and to emphasize that we have conducted the version of the experiment suggested by the reviewer. We have also conducted additional simulations to investigate another route to reduce overall presentation time of the image.

First, we would like to clarify that our protocol does control the presentation time: 1) the total presentation time of an image throughout the session is identical across participants and across images; and 2) the per-trial presentation time of the image with the cues is identical across trials, across images, and across participants. It is true that by default we present the images for a relatively long time, but that is because we aim to infer a segmentation map per

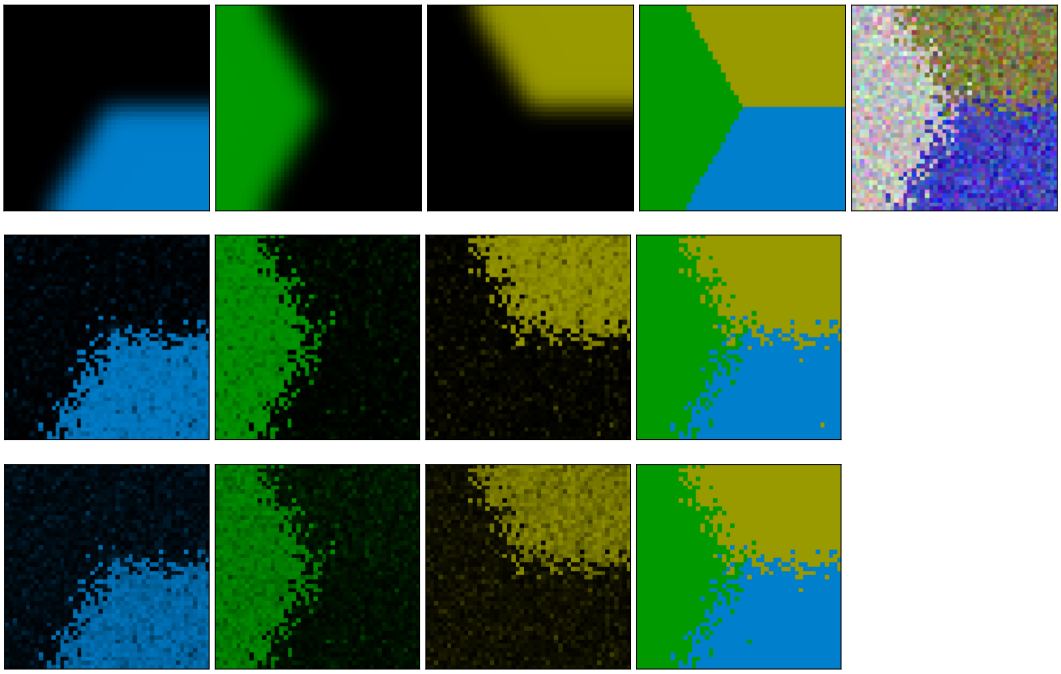

Figure 2: **Parametric model simulation**. The first two rows are reproduced from Fig. 8 in the manuscript. Last row is obtained with a quarter of the minimal number of trials required for reconstruction in the parametric case.

participant. Incidentally, the experiment duration is consistent with well-established protocols such as those that measure per-participant psychophysical kernels (Neri 2017; Ahumada Jr 1996; see also our response to point 3 below). We have added these clarifications in the revised text lines 583-586.

Second, as the reviewer correctly states, we can also run the experiment with just a few trials per participant per image, on a large number of participants, to reconstruct an aggregate segmentation map. We explain this more clearly on lines 178-186 and we show the results of this alternative way of running the experiment in Fig. 6 (natural images). This approach can be also taken to the extreme, by showing only one or a few trials per image and increase the number of participants and of tested images per participant. We addressed this in the Discussion (line 660).

Third, in the revised text (last paragraph of Discussion), we emphasize that the presentation time of the image required for a per-participant segmentation map can be drastically reduced with model-based reconstruction, if the model has fewer parameters than the minimal number of trials $(K-1)N^2$ required by the non-parametric method. To substantiate this point, we conducted additional simulations in which we reduced the number of measurements used for model-based reconstruction of the same texture image. The results are shown in Figure 2 of this response letter, where the first row are the ground truth segmentation maps and images, the second row is a reproduction of the results shown in the manuscript (Fig. 8) and the last row is the inference obtained when using a quarter of the minimal number or trials ($i.e.$ $\frac{(K-1)N^2}{4}$) required for reconstruction in the non-parametric case.

Rev 2 : c. There are few cases of inter-subject variability on an image-to-image basis in existing databases. That there are few cases of inter-subject variability on an image-to-image basis in extant databases is often true, but similarly the masks are often validated across subjects in smaller samples (as in the case of Cityscapes, with little variation in masks). In addition, if I have understood correctly, this is not an inherent advantage of the proposed

method, but a general comment on collecting segmentation masks. I do not see why more classic methods (like drawing) could not measure inter-subject variability at scale if it was deemed an important experimental question for the purpose. That most current benchmarks do not do this seems unrelated to the proposed methodology given that an actual benchmark of human segmentation masks is not put forth. A closer justification of the aforementioned issues in the paper is needed, as the paper's justification for the usefulness of its method is not currently appropriately justified.

We have revised the Introduction (lines 49, 55), Methods ("Detailed choices for each experiment"), and Discussion (lines 587-594) to clarify that the shortcoming of existing databases we refer to is the lack of measurement of variability in the map of the individual participant (intra-participant), not the variability between different participants (inter-participants). Measuring intra-participant variability is fundamental for understanding human perception, as documented by a long history of publications addressing perceptual uncertainty that we cite in the Introduction.

We agree with the reviewer that creating a new benchmark based on intra-participant variability is an important direction. This is indeed a line of research we are actively pursuing, but we believe it falls outside the scope of this manuscript whose main purpose is to validate and share the experimental method. We have added a sentence in the Discussion line 612.

We also agree with the reviewer that drawing/tracing approaches could be used to measure inter-participants variability systematically. In practice, it is also possible to measure intra-participant variability by asking the same participant to draw multiple times the segmentation of the same image. However, as discussed above (response to Points 2a,b), in either case this would not be purely a measure of perceptual uncertainty. For the intra-participant case, the time to collect masks would also increase linearly with the number of repetitions, as in our approach.

Rev 2 : 2. Following up on that, given that the paper claims problems with existing methods of segmentation map estimation, and claims to solve these problems, it seems important to me to have at least a small control experiment where this is quantified. How different are the segmentation maps in a free-drawing experiment using e.g. LabelMe (Russell et al., 2007) and the current method? Can between-subject uncertainty be estimated using traditional methods (this could potentially be tested by measuring the per-pixel variance across subjects)? I am not sure why the proposed method is superior in this respect given that it does not seem that within-subject variance is measured in e.g. the section starting at line 415. This need not be a separate large-scale experiment – I would be satisfied with seeing a small pool of participants complete a free-drawing task on the same images they segmented using the proposed method, downscaled to and evaluated at the same resolution as the proposed method. It would be important to me to know whether there are crucial perceptual factors or biases that this experiment can capture with its more controlled design than a typical free drawing task.

We thank the reviewer for the suggestion, that we have followed. At the end of all the experiments we asked the online participants to draw the maps they had used to solve the same/different task. We now show these drawings in Figure 6. Please see also the examples from BSD shown in Figure 3 of this letter, for a sense of the variability between participants when semantic categories are not pre-specified. More specifically, in the middle and bottom rows we observe that variability of segment numbers across participants in the BSD is captured by our experiment in the form of uncertainty of the map reconstructed from aggregate data (branches in front of the sky, see bottom row, 3rd image).

We would like also to emphasize that, as per our reply to point 1c above, our focus is on the variability of the segmentation map of each individual participant, not between different participants. In the revised manuscript lines 469-474 and 479-481, we have clarified that the Section

"Measured uncertainty in human participants correlates with image uncertainty" addresses the uncertainty of each individual participant. To further emphasize this, we now also show the segmentation and entropy map of each participant in Supplementary Figure 14. (Incidentally, we have also modified Figure 7 which was previously mistakenly showing bootstrapped results and not the average results over the participants.)

Lastly, we also report in supplementary Figure 14 the hand-drawn contours superimposed with the reconstructed entropy maps of each participant. There is no standard method to estimate uncertainty in contour reporting. Yet, the classical contour f-score used by Arbelaez *et al.* (Arbelaez et al. 2011) accounts for contour consistency. Therefore we measured the contour f-score of each participant with the ground truth contour and observed that contours are more consistent with the ground truth in the low uncertainty condition than in the high uncertainty condition. This however is inter-participants variability, not intra-participant.

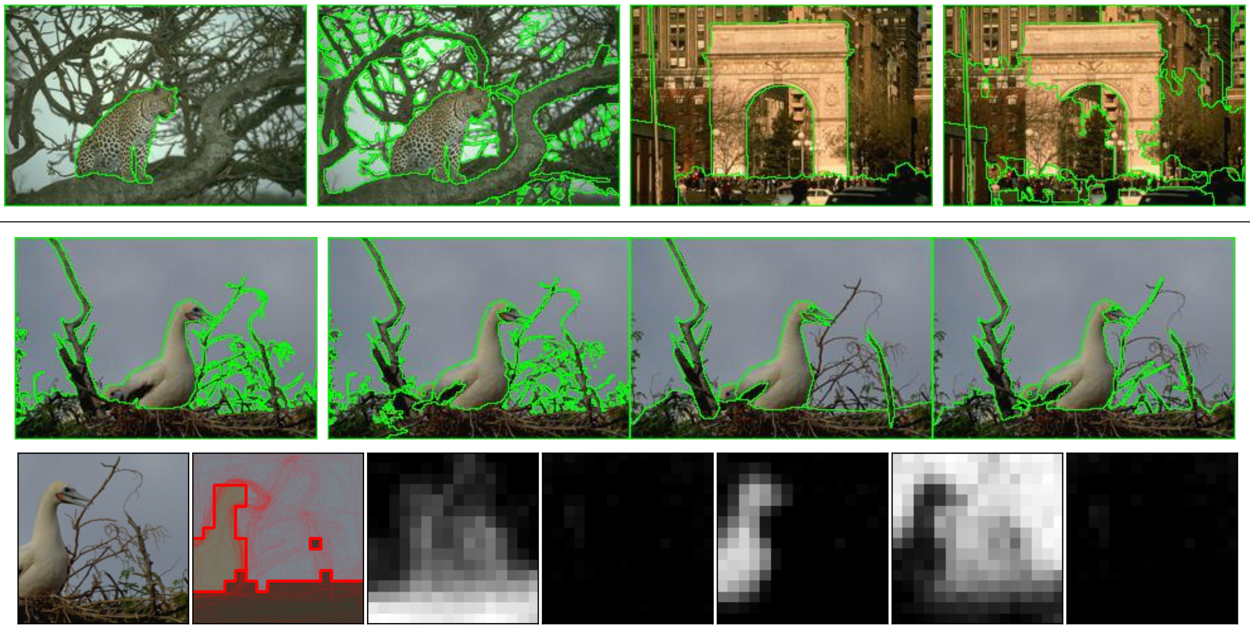

Figure 3: Top row: Examples of BSD images with contours drawn by human participants with varying level of details. Middle: Example of a BSD image with contours drawn by human participants. Bottom row: from our experiment, the hand-drawn contours of all participants (second map from left) and the measured probabilities (maps 3-7 from left). Note that contour variability observed across participants in BSD, is reflected in our measured probabilities, particularly the 3rd probability map for the segment that encompasses the branches.

Rev 2 : 3. I am concerned with the number of trials needed to estimate a segmentation mask, as well as with the resolution of the segmentation mask. According to the paper it takes a single participant approximately 50 minutes to measure the segmentation mask to a single stimulus at 11x11 resolution and 2 objects. I use the comparison of the CityScapes dataset again as it illustrates the point clearly: the CityScapes dataset reportedly takes approximately 90 minutes per image to segment, has a resolution of 1280x720 with up to 19 unique semantic classes, with potentially many instances of each. To collect high-quality segmentation masks with even a fraction of that resolution using the method presented in the paper, one would have to perform prohibitively expensive experiments to gather a database of any size for machine learning model evaluation, let alone training. Even small-scale psychophysical studies for stimuli of particular interest could prove very expensive.
Indeed, there are crucial scaling issues with the experimental procedure as it stands. The

number of trials needed scales quadratically with the resolution of the desired segmentation mask, and multiplicatively with the number of objects. This is in stark contrast to traditional drawing methods which scale approximately linearly with both. I think this substantially limits the potential of the method for naturalistic images of any complexity. This limitation should be further expanded upon in the paper.
As such, even if the method/paper is modified to answer the questions mentioned in the previous two points, I see its value almost entirely in measuring the validity of more traditional segmentation methods. I don't believe this issue is discussed in the paper, and I believe it should be, as it poses a strict limitation on the method's use cases. That being said, such a tool could be tremendously useful, and if the previous two comments can be answered, I believe this paper could meaningfully contribute to our understanding of the potential biases (or lack thereof) in measuring segmentation using more traditional, scalable methods.

We agree with the reviewer that our experiments take a long time (but see below for a more accurate calculation), which complicates its applicability in some important use cases, *e.g.* training data-hungry models. We have expanded the Discussion to be more explicit about this limitation and possible solutions (lines 655-664, see also Figure 2 of this response letter). On the other hand, there are valid reasons for the long duration, and there are numerous uses in which our method is applicable as is. We are also actively exploring several avenues to improve the scaling (see our reply to Point 1b). We have further revised the manuscript as follows.

First, we report more precisely the duration of the experiments (Methods section "Detailed choices for each experiment"). Reconstructing a deterministic map with K segments and N^2 pixels requires $(K-1)N^2$ trials, and each trial lasts typically around 1.2 seconds (300ms cues, plus 300ms stimulus and cues, which can be further reduced in on-site experiments, plus the reaction time which is on average 500-750ms for naive participants, see Figure 1). For a concrete example, the actual time to collect the mask for a 11x11 image with 2 segments, is a little over two minutes. This duration scales linearly with the number of repetitions (as would be the case with any other method, including manual tracing); if the goal is to measure the per-participant variability (*i.e.* probabilistic maps), 5-10 repetitions should be collected. In the original manuscript, we reported an average duration of 50 minutes with 10 repetitions for the experiments of Fig. 7, 9, but that included also voluntary breaks which cannot be controlled in the online experiments, and are longer than typical on-site experiments. This is now clarified in the revised text.

Second, we clarify that the duration of the experiment for characterizing the per-participant probabilistic maps, is in line with, and in fact often shorter than, comparable protocols used in traditional psychophysics. For instance, measuring per-participant psychophysical kernels (Neri 2017; Ahumada Jr 1996) often requires up to 40,000 trials and 50 hours of data collection for each participant. Relatedly, our method should not be seen simply as a competitor to, but rather as complementary to tracing-based approaches, because it allows to focus on perceptual factors (our reply to Point 1a) and to measure per-participant variability (our reply to Point 1c). Our results of Fig. 7 and 9, for instance, demonstrate that our method allows detailed characterization of segmentation uncertainty and model-based inference of the features used by the participants to segment the images. To emphasize this point, we have revised the text in the corresponding section (lines 479-481), and added a supplementary figure with the uncertainty maps of individual participants (Figure 14).

Third, we also thank the reviewer for the suggestion that one important use of our method is to understand the potential biases of tracing based methods (as discussed above). Relatedly, this would also be an opportunity to compare contour-based segmentation (as in tracing tasks, where the participants indicate whether a pixel is a boundary of a given object, rather than the segment label of each pixel) to region based segmentation (as in our method, where the

task is to compare the image regions around the two cues). We have added these uses in the Discussion (lines 638-652). Furthermore, we have expanded the Discussion to highlight two additional uses, of broad significance for understanding biological perceptual organization. Different from tracing tasks, our method employs a trial-based design, with precise control of cues and stimulus onset/offset. This facilitates analyzing and interpreting concurrent recordings of brain activity, *e.g.* with EEG, MEG or fMRI. Because the basic unit of our task is a simple discrimination, it may also be possible to train animal models on variants of our task, to study neural activity invasively in the relevant brain areas (*e.g.* early visual cortex and other areas involved in the decision process).

Rev 2 : Minor comments

Rev 2 : - Given that the per-image cost of segmenting is high, questions that relate to within-participant variability across stimuli seems difficult to assess. If one wants e.g. a database of 100 stimuli, this would effectively mean a single participant must perform 100 (or 800, if you want 5 objects!) sessions of the proposed experiment, which seems unreasonably difficult to accomplish in practice. This means that stimulus-specific variability and how that maps to perceptual variability (which is often what we are interested in as psychophysicists) can be difficult to measure using this methodology.

As discussed above, studying within-participant variability is a major focus of our method. Similar to many other well-established psychophysical methods, this requires studying a small number of stimulus conditions, in great detail (*i.e.* large number of trials).

Rev 2 : - Having to either fix the number of objects in the image beforehand, or having to collect a prohibitively large number of trials (in the paper, the example of a total of 4 hours of sessions per image per participant is given) appears to mean that one must curate the dataset of images to be segmented beforehand, and potentially impose large selection bias on the images that participants see. This could be especially problematic when one studies naturalistic images, where what counts as an independent object is often rather subjective and I worry that the method necessitates curating a dataset to exclude samples that could have many interpretations of the number of objects. This could lead to simpler or easier-to-segment images being selected for when making a benchmark, which could make human segmentation appear easier to explain using a model than it might really be.

We apologize for the lack of a detailed calculation of the session duration. As explained above, and in the revised text, the time required to collect data for a single image ranges from 2-30 minutes, allowing for several images in a single two-hour session on site. Conversely, for online experiments, the duration per image is less of a concern, when aggregating data across participants. This is now in Discussion (lines 655-664).

Regarding the issue raised here by the reviewer, that what counts as an independent object is often subjective, we believe our method allows the experimenter to study it quantitatively. This is beyond the scope of this paper, but for instance a straightforward approach would be to collect data for the same image, in both conditions (fixed K, and free K), and test if fixing K substantially reduces the variability across different participants. Analogously, as we speculated above in relation to Cityscapes, one could measure the maps with and without specifying the object categories to be segmented, and compare the variability across individuals.

Rev 2 : - The use of MAE as a metric to judge segmentation mask error seems odd. In most of the literature I am familiar with, metrics like IoU/ARI are used. This makes it difficult to judge the simulations for how good the results are in general to quickly gauge the accuracy of the method. If I look at Figure 12, bottom-right 64x64 segmentation mask, even at MAE=0.050 the segmentation mask looks clearly very inaccurate and would certainly be dubbed as a 'model failure' in the ML literature. While I haven't thought carefully about this, I wonder if comparing MAE across resolutions like this gives a meaningful measure of the

‘goodness’ of the segmentation mask.

The reviewer is correct that MAE should be interpreted differently from IoU/ARI. The revised text (Materials and Methods) now clarifies that our choice of MAE is because MAE is measured on probabilistic maps not on the deterministic segmentation mask. Specifically, the MAE is defined as the L^1 norm of the differences between the K-tuple of the ground truth and reconstructed probability at each pixel, averaged over pixels. Because it is measured on the probabilistic maps, the MAE reflects the accuracy of the estimation of uncertainty.

In Figure 12, the segmentation mask are inaccurate because the width of the kernel regularization (G) is fixed while the size of the image is changing. For that reason, the regularization is not the same when increasing the size of the image. We have added Figure 13 and text (lines 727-730) where we fixed this issue by adjusting the kernel width so that the regularization is done at a large scale. The segmentation mask is best recovered when accounting for the scale.

Rev 2 : Typos - Author summary: “contributions are three folds” = “contributions are threefold” - Line 134 and elsewhere in the document: “FIgure 6” = “Figure 6”

Corrected.

References

Neri, Peter (2017). “Object segmentation controls image reconstruction from natural scenes”. In: *PLoS biology* 15.8, e1002611.

Ahumada Jr, AJ (1996). “Perceptual classification images from Vernier acuity masked by noise”. In: *Perception* 25.1_suppl, pp. 2–2.

Arbelaez, Pablo et al. (2011). “Contour detection and hierarchical image segmentation”. In: *IEEE transactions on pattern analysis and machine intelligence* 33.5, pp. 898–916.
