# Supplementary material for: Measuring uncertainty in human visual segmentation
Source: PLoS Comput Biol. 2023 Sep 25;19(9):e1011483. doi: 10.1371/journal.pcbi.1011483 (PMC10553811; doi:10.1371/journal.pcbi.1011483)
Supplement: Attachment — Submitted filename: response_to_reviewers.pdf [file pcbi.1011483.s004.pdf]

Jonathan Vacher
Laboratoire MAP5 UMR 8145,
Université de Paris,
UFR Mathématiques & Informatique,
Campus Saint-Germain-des-Prés,
45 rue des Saints-Pères, 75006 PARIS

August 23, 2023

Dear Editor of PLoS Computational Biology journal and reviewers,

We would like to thank both reviewers again for their positive feedback. We appreciate that both reviewers considered that we have sufficiently addressed their concerns on the first round of review. We have accounted for the last details highlighted by reviewer 1 (see enclosed files, with modified text in blue) and have responded to his specific questions.

Sincerely,

Jonathan Vacher and co-authors

Encl: Detailed responses to reviewers. Edited manuscript with highlights. Edited manuscript.

Response to reviewer 1

Rev 1 : This version is certainly improved from the previous one and deals well with both my and the other reviewers' comments. Of course, last time I kvetched about math that I guessed the meaning of (generally correctly). It has been clarified, but on this reading I already knew what to expect. I have very few comments at this point.

Thank you for the evaluation and the additional feedback, please see our point by point replies below, and the correspondingly revised text in blue in the manuscript.

Rev 1 : * 205: I^2 is set of all pairs, but you obviously don't use pairs of identical points, but at no point in the math do you point that out. Unimportant.

We added this precision.

Rev 1 : * 243: I'm still tripped up by the proposition. You state in the proposition that the family of p-vectors paired with any one fixed grid square should be linearly independent (i.e., span N-space). But, which vectors are these. This is a proposition about estimating those vectors. So, do you mean the ground-truth set of vectors are linearly independent. Or, do you mean that the p_i in Eq. 6 are linearly independent, i.e., the sets of p_i you try when you minimize (i.e., you only consider sets of p_i such that all grid squares' associated sets of emerging vectors are linearly independent? Unobvious from the notation, because you use the notation p_i to denote ground truth AND to denote the parameters over which you maximize likelihood of the data. This confusion holds for the proof in S1 as well (do you minimize only over sets that satisfy linear independence?).

The families of vectors that need to be linearly independent are the ones that are critical points of the loss functions ℓ and ℓ_s. We added this precision in the proposition and in the proof. The ground truth does not really matter because, in practice, we are never really finding the ground truth as a solution of our optimization problem.

Rev 1 : * I like switching from independent to linearly independent in the text and would ask that you do so consistently. You missed 258, 286, 290, also the next to last line in S1

Done.

Rev 1 : * 259: verified → obtained???

Done.

Rev 1 : * 315: problem → Equation?

Done.

Rev 1 : * 317: By "periodic" convolution do you mean one that wraps around the image edges? If so, why?

Thanks for pointing that out. This is a mistake, here we use the standard discrete convolution. We have edited accordingly and added a footnote about how the edges are handled.

Rev 1 : * 436: "Similar to" or "lower than" than that of a kernel? In fact, what do you mean by the dominant spatial frequency of a Gaussian, since that's a low-pass filter with dominant frequency that is always zero?

You are absolutely right. Thanks for pointing out this factual mistake. We now talk about the range of spatial frequency.

Rev 1 : * S1: I don't understand the if-and-only-ifs in the proof. I'm sure it stems from linear independence, but couldn't work it out. You might clarify if you think that's appropriate.

A definition of linear independence of a family of vectors $(x_1, \dots, x_n)$ is

$$\forall (\lambda_1, \dots, \lambda_n) \in \mathbb{R}^n, \ \sum_{i=1}^{n} \lambda_i x_i = 0 \quad \Rightarrow \quad \lambda_1 = \dots = \lambda_n = 0.$$

This is what we use to conclude that all scalar coefficients scaling the vectors $p_{\mathbf{i}}$ are equal to zero and hence that $p_{\mathbf{i}} \cdot p_{\mathbf{j}} = k_{\mathbf{i},\mathbf{j}}$. We added one equivalence to clarify the relation.

Rev 1 : * S2: Texture synthesis, line 3: There's a missing citation/latex bug
Fixed.

Response to reviewer 2

Rev 2 : I believe that the authors' changes have made the manuscript stronger and congratulate them on an excellent manuscript. The authors have addressed all my comments and I fully support its publication.

Thanks you again for your feedback !
